# The Mechanism of Environmental Endocrine Disruptors (DEHP) Induces Epigenetic Transgenerational Inheritance of Cryptorchidism

**DOI:** 10.1371/journal.pone.0126403

**Published:** 2015-06-02

**Authors:** Jinjun Chen, Shengde Wu, Sheng Wen, Lianju Shen, Jinpu Peng, Chao Yan, Xining Cao, Yue Zhou, Chunlan Long, Tao Lin, Dawei He, Yi Hua, Guanghui Wei

**Affiliations:** Department of Pediatric Research Institute, Children's Hospital of Chongqing Medical University, Ministry of Education Key Laboratory of Child Development and Disorders, Chongqing, China; Clermont Université, FRANCE

## Abstract

Discussion on the role of DEHP in the critical period of gonadal development in pregnant rats (F0), studied the evolution of F1-F4 generation of inter-generational inheritance of cryptorchidism and the alteration of DNA methylation levels in testis. Pregnant SD rats were randomly divided into two groups: normal control group and DEHP experimental group. From pregnancy 7d to 19d, experimental group was sustained to gavage DEHP 750mg/kg bw/day, observed the incidence of cryptorchidism in offspring and examined the pregnancy rate of female rats through mating experiments. Continuous recording the rat’s weight and AGD value, after maturation (PND80) recording testis and epididymis’ size and weight, detected the sperm number and quality. Subsequently, we examined the evolution morphological changes of testicular tissue for 4 generation rats by HE staining and Western Blot. Completed the MeDIP-sequencing analysis of 6 samples (F1 generation, F4 generation and Control). DEHP successfully induced cryptorchidism occurrence in offspring during pregnancy. The incidence of cryptorchidism in F1 was 30%, in F2 was 12.5%, and there was no cryptorchidism coming up in F3 and F4. Mating experiment shows conception rate 50% in F1, F2 generation was 75%, the F3 and F4 generation were 100%. HE staining showed that the seminiferous epithelium of F1 generation was atrophy and with a few spermatogenic cell, F2 generation had improved, F3 and F4 generation were tend to be normal. The DNA methyltransferase expression was up-regulated with the increase of generations by Real Time-PCR, immunohistochemistry and Western Blot. MeDIP-seq Data Analysis Results show many differentially methylated DNA sequences between F1 and F4. DEHP damage male reproductive function in rats, affect expression of DNA methyltransferase enzyme, which in turn leads to genomic imprinting methylation pattern changes and passed on to the next generation, so that the offspring of male reproductive system critical role in the development of imprinted genes imbalances, and eventually lead to producing offspring cryptorchidism. This may be an important mechanism of reproductive system damage.

## Introduction

Epigenetic transgenerational inheritance involves the germline transmission of an altered epigenome and phenotypes across generations in the absence of direct environmental exposures[1]. The germline epigenome undergoes reprogramming during fetal gonadal development[2]. Environmentally induced germline epigenetic modifications can occur during DNA demethylation and remethylation period and become permanently programmed similar to the DNA methylation of an imprinted gene[3]. The male germline propagates this epigenetic change after fertilization to all somatic cells resulting in an alteredepigenome and transcriptome that may lead to cryptochidism in future generations[4]. A number of environmental chemical exposures have been shown to promote epigenetic transgenerational inheritance of cryptochidism and the transgenerational epigenetic changes may be used as biomarkers of exposure and disease[5]. This study was designed to investigate the potential that dioxin ((Di(2-Ethylhexyl) Phthalate, DEHP) promotes epigenetic transgenerational inheritance of cryptochidism. In mammals DEHP has a half-life of hours and rise fetal death rate,fetal abnormalities,weight loss,causes liver and kidney deseases[6]. The diseases associated with exposure to DEHP include cryptorchidism and hypospadias[7]. The majority studies have focused on fetal exposures, a study of DNA methylation level of genomes in the mouse testis prompt that exposure to DEHP during pregnancy increases the DNA methylation level of the genome in the testis of the offspring and affects the modification of the genome, which may be one of the important causes of the lesion in the reproductive system[8]. No human studies have investigated transgenerational (4 generations) effects of DEHP. Animal models have been used to study the toxicological effects of DEHP. DEHP has been shown to produce reproduction toxity in newborn rats[6]. Adverse effects in animals include developmental neurobehavioral effects, developmental reproductive (sperm counts, urogenital malformations) effects and immune toxic effect[6, 9]. Previous studies with DEHP used high dose(500 to 750mg/kg bw/day) and only evaluated the direct exposure of adult and fetus (F0 and F1) generations[10]. The current study used 750 mg/kg bw/day dose mixture of DEHP and corn oil. However, it was designed to investigate the potential that DEHP may promote transgenerational disease. Since the exposure of a gestating F0 generation female also directly exposes the F1 generation fetus and germ line that will generate the F2 generation, then F3 generation without direct exposure[11].

## Materials and Methods

### Ethics statement

All experiments involving animals were in accordance with the Guide for the Care and Use of Laboratory Animals and accredited by the Institutional Animal Care and Use Committee (IACUC). The protocol was approved by the Association for Assessment and Accreditation of Laboratory Animal Care International, China and Experimental Animal Committee of the Chongqing Medical University (license numbers: SCXK (Yu) 2012–0001 and SYXK (Yu) 2012–0001).

### 1. Animals and drug delivery system

SD female and male rats of an outbred strain were maintained in ventilated (up to 40 air exchanges/hour) isolator cages (with dimensions of 60cmW×40cmD× 20cmH) containing sterilized chips (pinewood shavings) as bedding, on a 14h light and 10h dark regimen, at a temperature of 25±1°C and humidity of 40% to 70%. The noise below 85 decibels, ammonia concentration of 20 parts per million. Rats were fed with standard rat diet and distilled water for drinking. At pro-estrus as determined by daily vaginal smears, the female rats (80 days of age) were pair-mated with male rats (80 days), at least their weight were more than 250g. On the next day, the pairs were separated and vaginal plug was founded that female rats were tentatively considered pregnant (gestation day, GD1). Monitoring of vaginal smears was continued for diestrus status in these rats until day 7. Pregnant rats for the treatment group were given daily intragastric administration of DEHP and corn oil mixture (750 mg/kg bw/day, Yuanye Bio-technology Co, Ltd, Shanghai,China) and an equal volume of pure corn oil (Sigma) on days GD7 untill GD19 of gestation. DEHP was dissolved in corn oil. Pregnant rats for the control group were given daily intragastric administration of pure corn oil (750 mg/kg bw/day). The pregnant female rats treated with DEHP were designated as the F0 generation. All experimental protocols for the procedures with rats were accredited by the Association for Assessment and Accreditation of Laboratory Animal Care International, China and Experimental Animal Committee of the Chongqing Medical University (license numbers: SCXK (Yu) 2012–0001 and SYXK (Yu) 2012–0001).

### 2. Raising F1, F2, F3 and F4 generations

After intragastric administration, at the GD21 or GD22 F0 generation rats give birth to children, their offsring was F1 generation. Randomly selected eight male F1 after normal feeding to 80 days old, mating with normal female SD rat then breed F2 generation, and so on. The control group is normal breeding will not be any processing. No sibling or cousin breeding was performed so as to avoid inbreeding. Note that only the original F0 generation pregnant females were intragastric administration with DEHP.

### 3. Cryptorchidism case and histology of reproductive system

A. Cryptorchidism determine: F1-F4 generations (80 days after birth, PND80) under the condition of not less than 26°C environment temperature, testis has not been observed in bilateral or singal scrotal three times was considered cryptorchidism. B. Sperm counts: At PND80 aquaired epididymis, cut into pieces after weighing, placed 5 min in 5 ml warm saline to release sperm, by two layer lens paper filtration, record the filtered volume. Diluted with warm saline water, white blood cell count plate count, according to count the sperm density, calculate the total number of sperm in the filtrate, divided by the total epididymal sperm is the epididymal sperm count per gram in weight. Sperm of smear and Eosin staining can be used to observe sperm shape abnormality.

### 4. DNA methylation transferase mRNA and protein expression

A. Dnmt1, Dnmt3a and Dnmt3b mRNA expression of detection: design specific primers (Table 1), using ABI fluorescence quantitative PCR Stepone test three methylation transferase mRNA expression between groups. B. Dnmt1, Dnmt3a and Dnmt3b protein expression of detection: using Western blot detection of every generation of testicular tissue in three kinds of protein expression level of DNA methylation transferase and their specific antibody. (respectively: the article number of sc-20701, sc-20703, sc-20704, Santa Cruz).

**Table 1:** Quantitative real-time PCR Primers for DNA methytransferases and β-actin.

Gene	Account number	Primer	Size in bp
Dnmt1	NM_010066.3	Forward:5’-ACTCCCTTCGGGCATAGCAT-3’	148
		Reverse:5’-AGGTTGCAGACGACAGAACAG-3’	
Dnmt3a	NM_007872.4	Forward:5’-CTCTCGACTGTTCTCCTGCTG-3’	160
		Reverse:5’-GTAGACCCACGGTGACTTGTA-3’	
Dnmt3b	NM_001003960.3	Forward:5’-GACGTCGAGCATCATCTTCA-3’	197
		Reverse:5’-AACTGATGGGGTACTGACGC-3’	
β-actin	NM_007393	Forward:5’-AGAGGGAAATCGTGCGTGAC-3’	138
		Reverse:5’-CAATAGTGATGACCTGGCCGT-3’	

### 5. Study of reproductive function (mating experiment)

Randomly selected from each group of 8 male young rats fed to sexual maturity (60 days after birth, PND60), every male rats pair-matting with a randomly selected female rat of two weeks (about three sexcycles), observing the conception rate of female rat in each group.

### 6. Measuring the distance between the anus and genitals (AGD)

After intraperitoneal injection of 10% chloral hydrate anesthesia animal anatomy, testis and epididymis were separated and weighing. Part of MDF fixed, and the rest were reserved in -80°C refrigerator frozen storage. Routine paraffin section (4μm), after fully fixed dewaxing, xylene through the high concentration to low concentration alcohol dehydration in turn, transparent, wood grain, eosin staining. In 1,3,7,14,21,30 day, measuring the distance of anus to genital.

### 7. DNA Extraction and Methylated DNA Immunoprecipitation (MeDIP)

DNA was isolated using the Trizol method per the manufacturer’s protocol, from the same testes Trizol preparations that were used for RNA isolations. Therefore, three independent DNA Trizol fractions from testes per group were used to obtain three different biological replicates of DNA samples from each of the two treatment groups. Each of these DNA samples were then used for methylated DNA immunoprecipitation (MeDIP). DNA was independently extracted from 2 generations of testes (F1 and F4). MeDIP Sequencing for 6 Rat Samples, F1×3 and F4×3. The mRNA processing and hybridizations were performed at the Laboratory For Urogenitaldevelopment & Anomalies, Chidren’s Hospital of Chongqing Medical University in China. Using standard Promega Corporation reagents and protocols. Briefly, mRNA was reverse transcribed into cDNA with random primers, then cRNA was transcribed from the cDNA, and from that, singlestranded sense DNA was synthesized which was fragmented and labeled with biotin. Biotin-labeled, fragmented ssDNA was then data generated from Illumina HiSeq 2000 (Kangchen MeDIP Sequencing Service, CHINA). We have completed the MeDIP-sequencing analysis of the 6 samples. DNA samples were fragmented to a size range of ~200-900bp with a Diagenode Bioruptor. About 800ng of fragmented DNA was prepared for Illumina HiSeq 2000 sequencing as the following steps: 1) End repair of DNA samples with T4 DNA polymerase, Klenow DNA polymerase, and T4 PNK; 2) A single ‘A’ base was added to the 3' ends with Klenow (exo minus) polymerase; 3) Ligation of Illumina's genomic adapters to DNA fragments; 4) MeDIP to enrich methylated DNA by anti-5-methylcytosine antibody; 5) PCR amplification to enrich precipitated fragments; 6) Gel purification to extract ~300–1000 bp DNA fragments. The completed libraries were quantified by Agilent 2100 Bioanalyzer. The libraries were denatured with 0.1 M NaOH to generate single-stranded DNA molecules, captured on Illumina flow cell, amplified *in situ*. The libraries were then sequenced on the Illumina HiSeq 2000 following the TruSeq SBS Kit v5 protocol. After sequencing images generated, the stages of image analysis and base calling were performed using Off-Line Basecaller software (OLB V1.8). After passing Solexa CHASTITY quality filter, the clean reads were aligned to rat genome (UCSC RN5) using BOWTIE software (V2.1.0). About 64.2 million mapped reads which represented over 6.4 billion bases of sequence were obtained for all the samples. A methylation score for any region in the genome was defined as number of reads per kb (A.K. Maunakea et al, 2010). The signal profiles (at 50 bp resolution) in UCSC WIG file format were generated from MeDIP-seq data, which can be visualized using UCSC genome browser or IGB browser (Integrated Genome Browser, Java Runtime Environment needed, http://www.bioviz.org/igb/).

### 8. Pathway and gene network analysis

Known functional relationships among the F4 generation differentially expressed genes were identified using the KEGG pathways from the University of Kyoto (Japan) Encyclopedia for Genes and Genome website (http://www.genome.jp/_eg/) and Pathway Express (http://vortex.cs.wayne.edu)[12]. Pathway analysis is a functional analysis mapping genes to KEGG pathways. The p-value (EASE-score, Fisher-Pvalue or Hypergeometric-Pvalue) denotes the significance of the Pathway correlated to the conditions. Lower the p-value, more significant is the Pathway. (The recommend p-value cut-off is 0.05), using an unbiased, automated survey of published scientific literature (Global Literature Analysis). This analysis identifies functional relations among genes, such as direct binding, up-regulation or down-regulation and also builds subnetworks of genes and cellular processes based on their interconnections.

### 9. Statistical analysis

Using SPSS 17. 0 software for statistical analysis, data representation x±s, P < 0.05 to have statistical significance.

## Results

1. After DEHP gavaged pregnant F0, F1 generation has 30% incidence of cryptorchidism, F2 generation of 12.5% incidence of cryptorchidism, F3 and F4 generations not seen cryptorchidism occurred (Table 2). Results show DEHP can lead to "acquired" children cryptorchidism inter-generational genetic phenomenon, with the increase of generations, offspring of male rats gradually reduce the incidence of cryptorchidism.

**Table 2:** DEHP induced F1-F4 incidence of cryptorchidism.

Group	Pregnant Rats(pcs)	Total Number Born(pcs)	Male litter size(pcs)	Cryptorchidism several(pcs)	Cryptorchidism Ratio(%)
Control	8	86	47	0	0
F1	4	26	10	3	30*
F2	6	45	16	2	12.5*
F3	8	71	32	0	0
F4	8	69	38	0	0

* compared with control group, P<0.05

2. DEHP induced offspring of male rat reproductive function damage inspection as shown in (Table 3), control males makes the conception of female was 100%, the ratio of DEHP processing makes the conception rate of 50% in F1, F2 generation was 75%, F3 and F4 generation were 100%. This shows DEHP in offspring of male rat reproductive function damage degree gradually decline.

**Table 3:** DEHP induced male rat reproductive function damage.

Group	Female(pcs)	Pregnant Rats(pcs)	Pregnant Ratio(%)
Control	8	8	100
F1	8	4	50
F2	8	6	75
F3	8	8	100
F4	8	8	100

* compared with control group, P<0.05

3. DEHP induced rats’ (F1-F4 generations) weight and anogenital distance(AGD) value change and the histology of the testis and epididymis results: 1) compared with control group,DEHP group’s weight significantly decreased, especially to the F1, F1-F2 generation of AGD value also decreased significantly (Table 4).

**Table 4:** DEHP influence male rat’s weight and AGD.

	PND1		PND3		PND7		PND14		PND21		PND30	
	weight (g)	AGD(mm)	weight (g)	AGD(mm)	weight (g)	AGD (mm)	weight (g)	AGD(mm)	weight (g)	AGD(mm)	weight (g)	AGD(mm)
Control	7.95±0.13	5.67±0.20	9.6±0.50	6.75±0.82	15.04±0.39	7.19±0.46	30.72±3.12	11.27±0.92	45.17±4.56	16.67±0.48	75.01±7.52	25.44±1.15
F1	6.14±0.73*	4.68±0.69*	7.91±0.96*	5.31±0.80*	13.36±2.64*	6.79±0.92*	27.38±3.06*	10.62±0.69*	42.89±3.92	14.67±1.16*	60.65±7.42*	19.84±1.54*
F2	6.58±0.53*	3.98±0.66*	8.18±0.87*	5.32±0.67*	13.88±2.46*	6.57±0.66*	26.61±3.73*	9.97±0.79*	42.66±4.87	15.34±1.16	67.17±8.93	20.91±1.48*
F3	7.09±0.49	5.21±0.44	9.60±0.50	6.55±0.36	17.25±4.48	7.88±1.14	33.17±8.85	11.07±1.73	49.3±15.89	16.82±1.49	68.81±17.06*	20.76±3.46*
F4	7.11±0.21	4.88±0.61	10.03±0.48	6.38±0.22	18.21±1.34	8.02±0.76	31.08±2.79	10.86±5.03	51.09±3.79	15.86±2.31	70.23±2.01	21.08±0.58

* compared with control group, P<0.05

4. Weigh F1-F4 generations testis and epididymis(PND80), significantly lower than the normal control group, the difference was statistically significant (P < 0.05) (Table 5).

**Table 5:** DEHP influence male rat’s testis and epididymis.

Group	Testis weight(g)	Epididymis weight(g)
Control(8pcs)	1.90±0.28	0.63±0.05
F1(10pcs)	1.55±0.19	0.44±0.11#
F2(16pcs)	1.61±0.11*	0.45±0.05#
F3(32pcs)	1.84±0.13	0.61±0.10
F4(38pcs)	1.87±0.75	0.63±0.38

* ^#^ compared with control group, P<0.05

**Fig 1:**
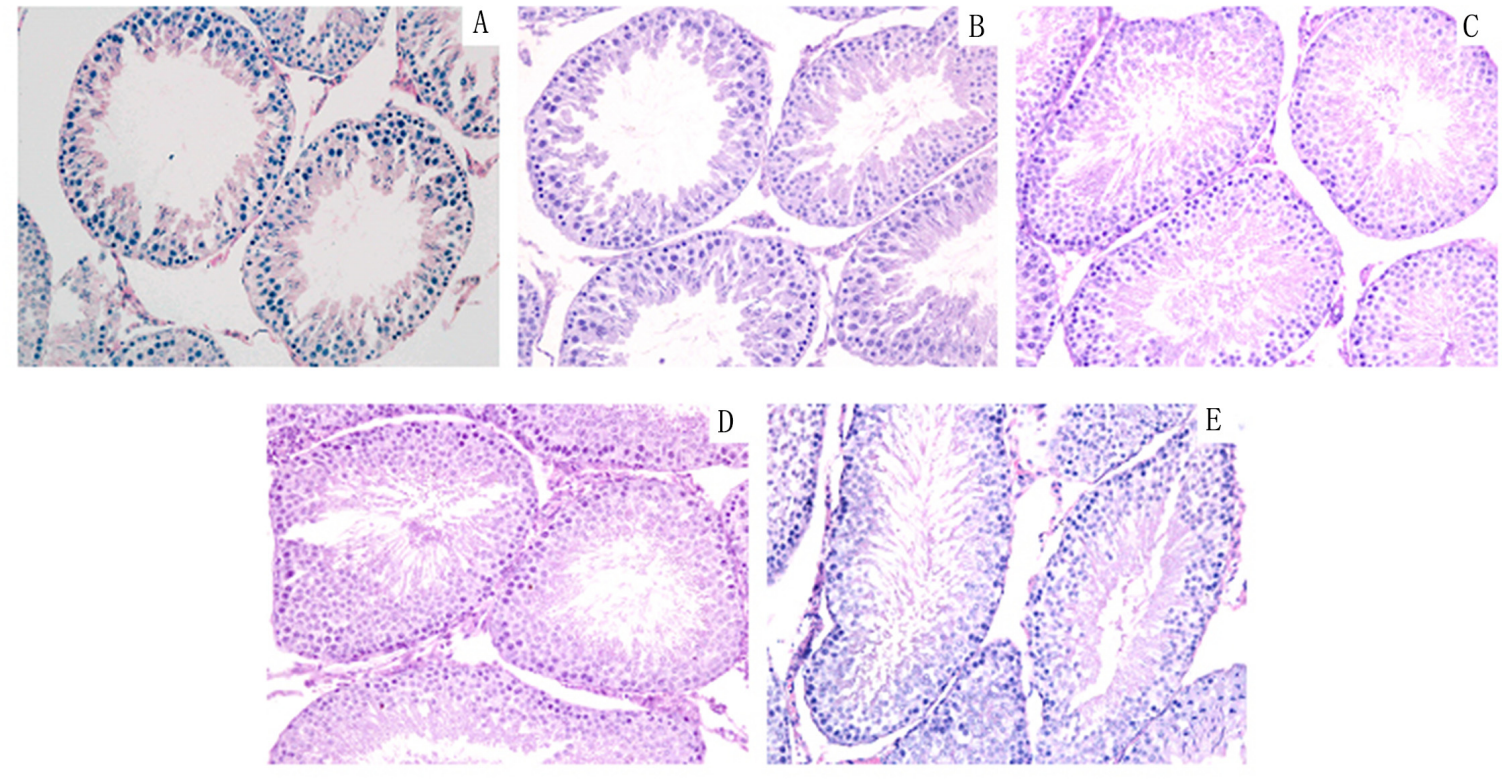
Control group and DEHP experimental group offspring (F1-F4) male rat’s testicular histology expression. A:F1;B:F2;C:F3;D:F4 E: Control (200×).

**Fig 2:**
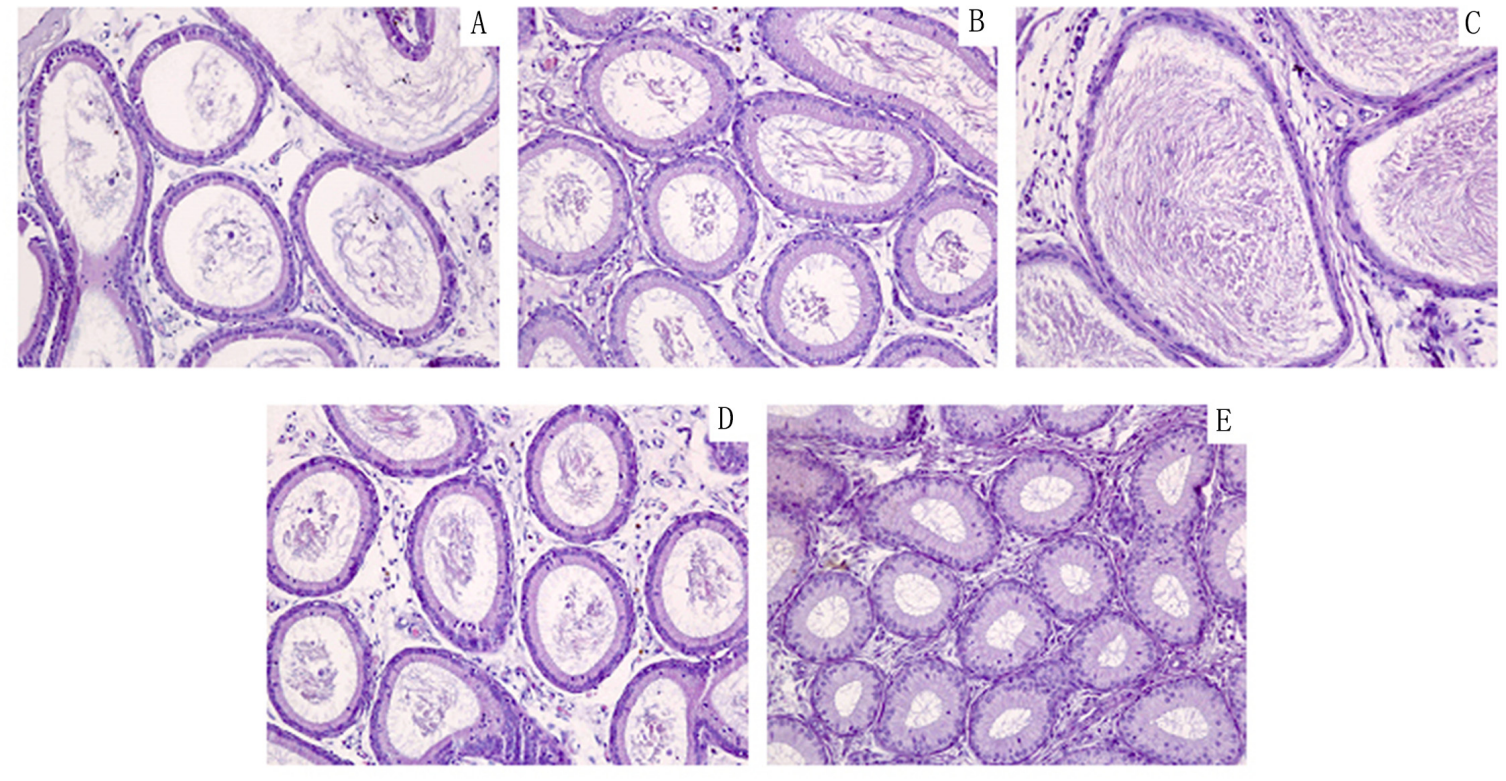
Control group and DEHP experimental group offspring (F1-F4) male rat’s epididymis histology expression. Normal control group of testicular seminiferous tubule epithelial developed normally, we can observe each stage sperm cells and a large number of sperm cells in the epididymis pipe. DEHP experimental F1 generation observed seminiferous tubule epithelial obvious atrophy, less sperm cells, pathologic hyperplasia of interstitial cells, number of sperm in the epididymis lumen scarce or lack, luminal epithelium cell falls off. F2 generation of rat testis partial seminiferous tubule epithelium atrophy, sperm cells less; F3 and F4 generation were tend to be normal, sperm cells grading well. A:F1;B:F2;C:F3;D:F4 E: Control (200×).

In order to further observe the offspring of male rat reproductive organs injury by DEHP,HE staining to observe histomorphology of testis and epididymis. (Figs 1 and 2).

Due to previous results show DEHP processing result in offspring reproductive function of male rats (Table 3), we hypothesized that may be due to the decrease in the number ofsperm and/or sperm deformity rate increase. Therefore, we tested the offspring number of sperm in male rats epididymis and sperm deformity rate. Sperm count results show that the F1 generation of sperm counts than normal control group decreased significantly (P<0.05). F3 and F4 generation compared with the normal group there was no significant difference (P>0.05) (Table 6).

**Table 6:** DEHP influence male rat’s sperm counts.

Group	Sperm counts (×10^6^ /g)
Control(8pcs)	80.03±8.35
F1(10pcs)	15.18±7.95*
F2(16pcs)	52.64±24.46
F3(32pcs)	75.43±8.68
F4(32pcs)	80.48±2.56

* compared with control group, P<0.05

**Fig 3:**
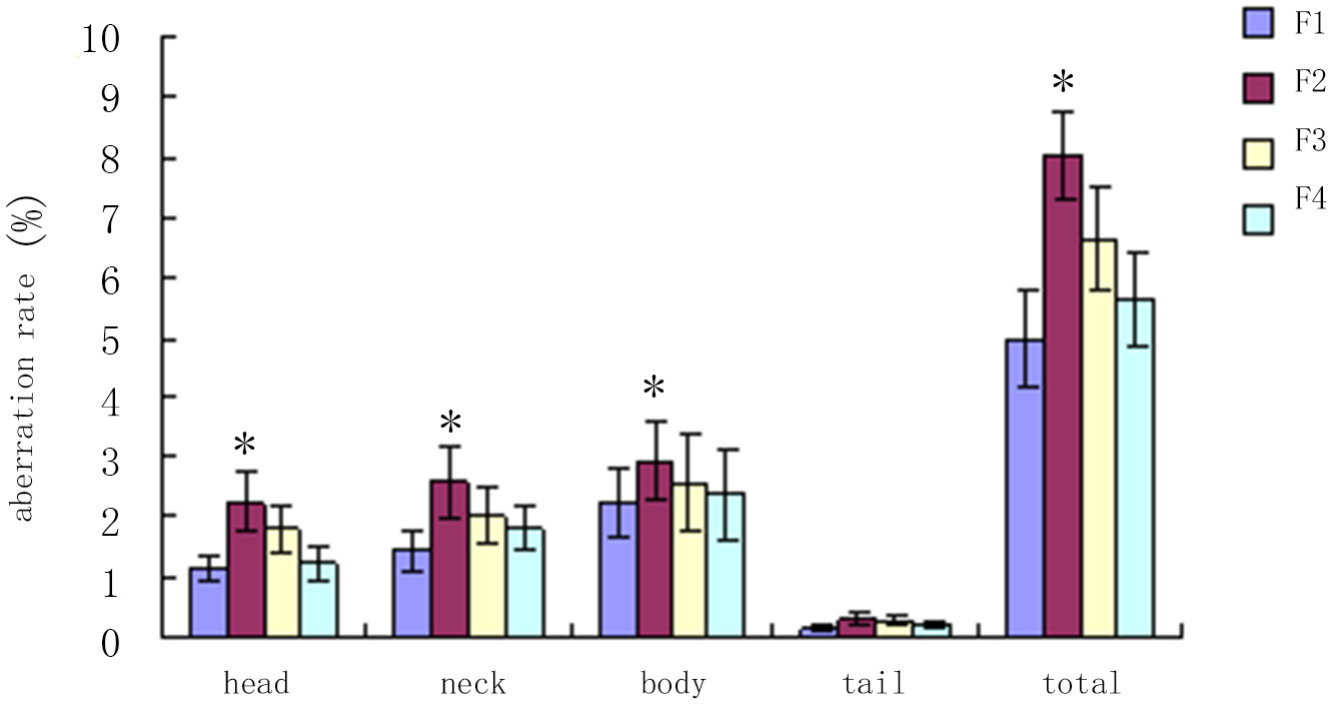
F1-F4 semen quality comparison. * compared with control group, P<0.05.

**Fig 4:**
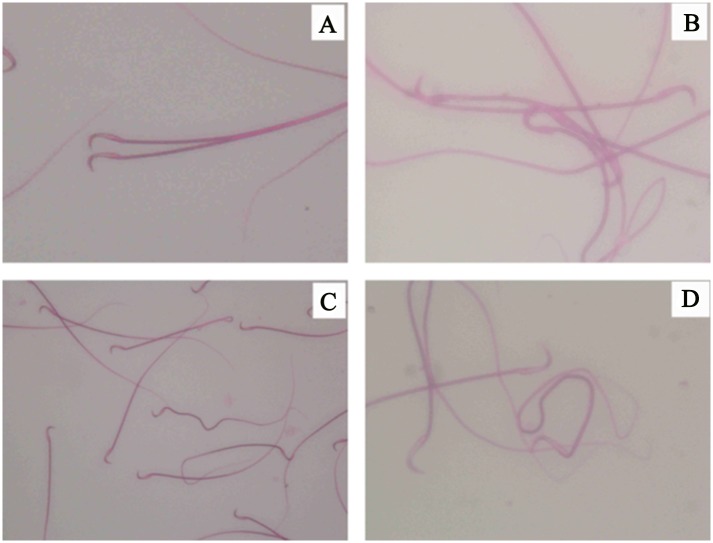
Sperm abnormality. This is illustrated by the results of the most serious damage of F1 generation of male rats’reproductive function, and with the increase of genetic algebra, damage degree showed a trend of decline. A:double head;B:head;C:curve;D:fold.

Sperm deformity rate exanmination by sperm smear staining showed the F1 is significantly higher than normal group (P<0.05), the head and neck deformity more significantly (P<0.05). F3 and F4 generation sperm deformity rate compared with normal group, there was no significant difference (P>0.05) (Table 7, Figs 3 and 4).

**Table 7:** DEHP influence male rat’s semen quality (aberration rate).

	Control(8 pcs)	F1(10 pcs)	F2(16 pcs)	F3(32 pcs)	F4 (32 pcs)
Head	1.14±0.23	2.25±0.51*	1.79±0.39	1.23±0.28	1.01±0.39
Neck	1.43±0.34	2.57±0.58*	2.03±0.47	1.82±0.36	1.34±0.12
Body	2.24±0.56	2.91±0.64	2.56±0.82	2.37±0.75	1.56±0.06
Tail	0.16±0.05	0.32±0.09	0.27±0.08	0.21±0.05	0.26±1.03
Total	4.97±0.83	8.05±0.72*	6.65±0.86	5.63±0.79	4.27±0.82

* compared with control group, P<0.05

5. By Real Time PCR and immunohistochemistry and Western Blot, we detect male rats’ testis DNA methyltransferase, including Dnmt1, Dnmt3a and Dnmt3b mRNA and protein expression levels (Table 8). After dealing with the DEHP, progeny F1 and F2 generation of male rats’ testis of three kinds of DNA methyltransferase, Dnmt1, Dnmt3a and Dnmt3b mRNA expression level significantly raised (p < 0.05), and its expression in F3 generation of testicular relatively normal, has no statistical significance compared with the group.

**Table 8:** Three kinds of DNA methyltransferase mRNA expression.

	Control(2)	F1(2)	F2(2)	F3(2)	F4(2)
Dnmt1	1.0738±0.6452	2.6841±0.2461*	1.8062±0.2664*	1.2633±0.2138	1.001±0.0875
Dnmt3a	1.1176±0.3856	17.4136±1.2648*	11.5486±1.0643*	1.3647±0.2100	1.1031±0.2981
Dnmt3b	1.0891±0.2103	2.8645±1.1032*	2.6144±0.9834*	1.1852±2.1264	1.1087±0.1056

* compared with control group, P<0.05

**Fig 5:**
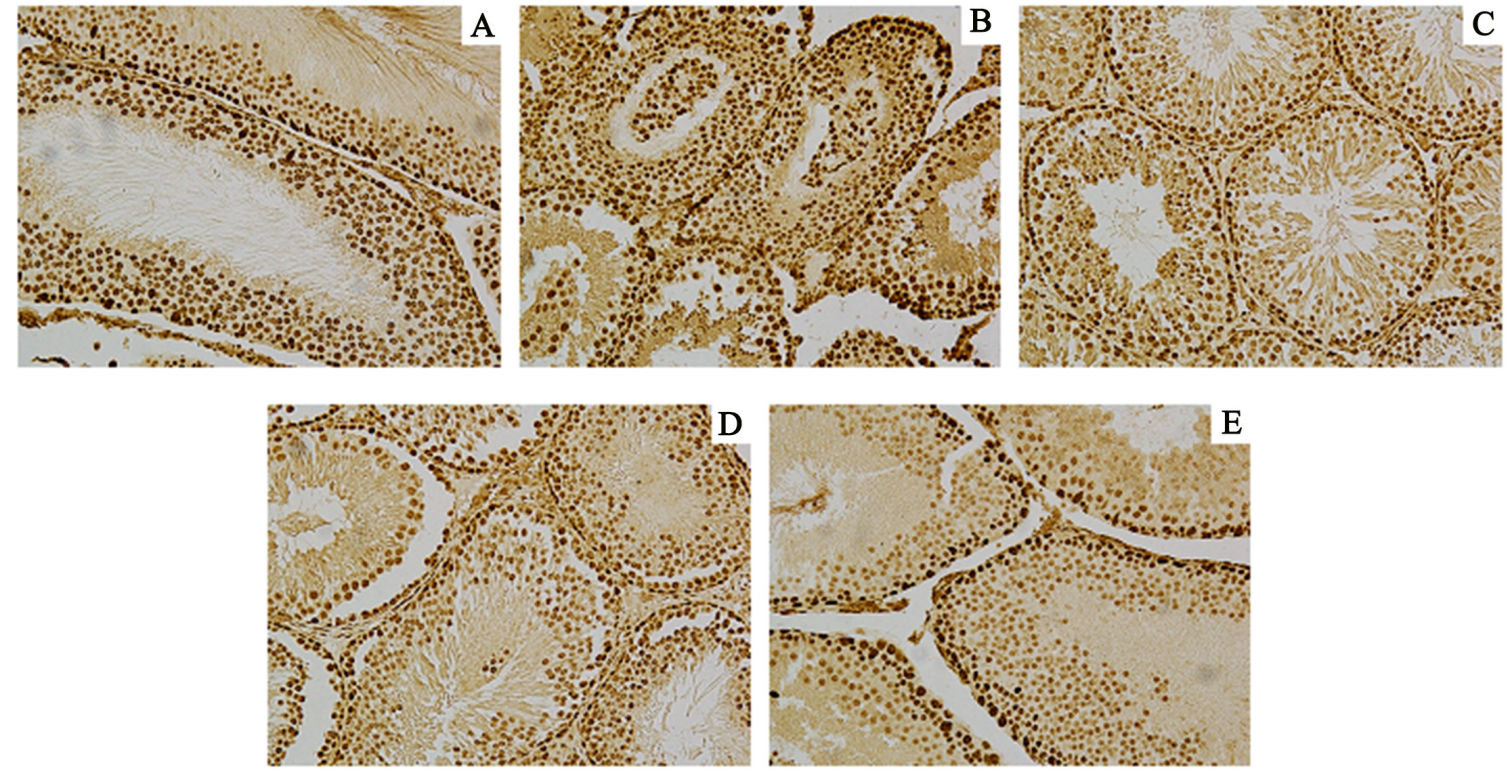
Control group and DEHP experimental group offspring (F1-F4) male rat’s DNMT1 expression. High expression of dnmt1 in the F1, F2 generation significantly reduced, F3 and F4 generation basic without expression. A:Control;B:F1;C:F2;D:F3 E:F4 (×200)

**Fig 6:**
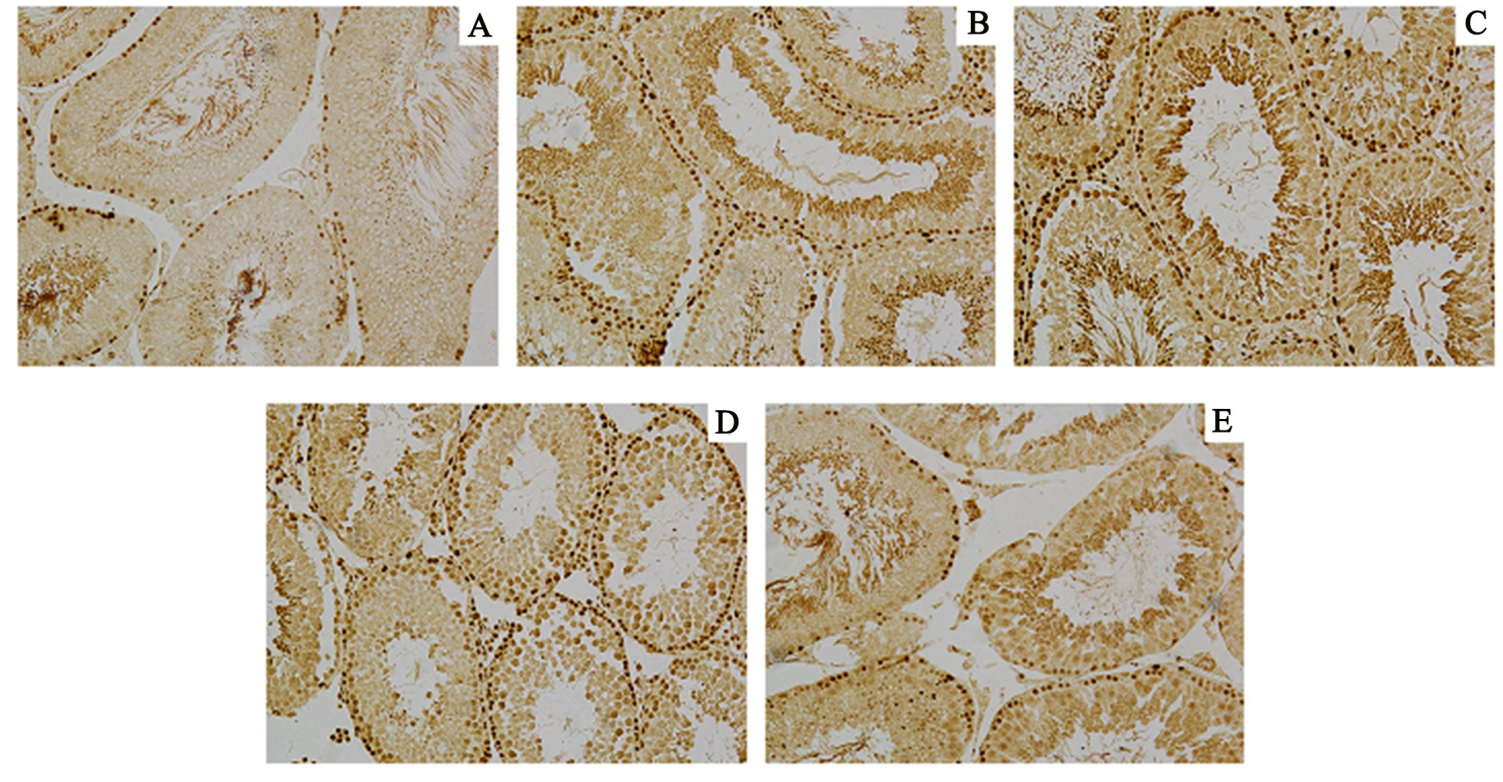
Control group and DEHP experimental group offspring (F1-F4) male rat’s DNMT3a expression. High expression of dnmt1 in the F1 and F2 generation, F3 significantly reduced and F4 generation basic without expression. A:Control;B:F1;C:F2;D:F3 E:F4 (×200)

Immunohistochemical results show F1 and F2 generation testicles Dnmt1 protein expression levels were significantly higher than control group (Fig 5), and the protein expression level of Dnmt3a rise only in the F1 (Fig 6).

**Fig 7:**
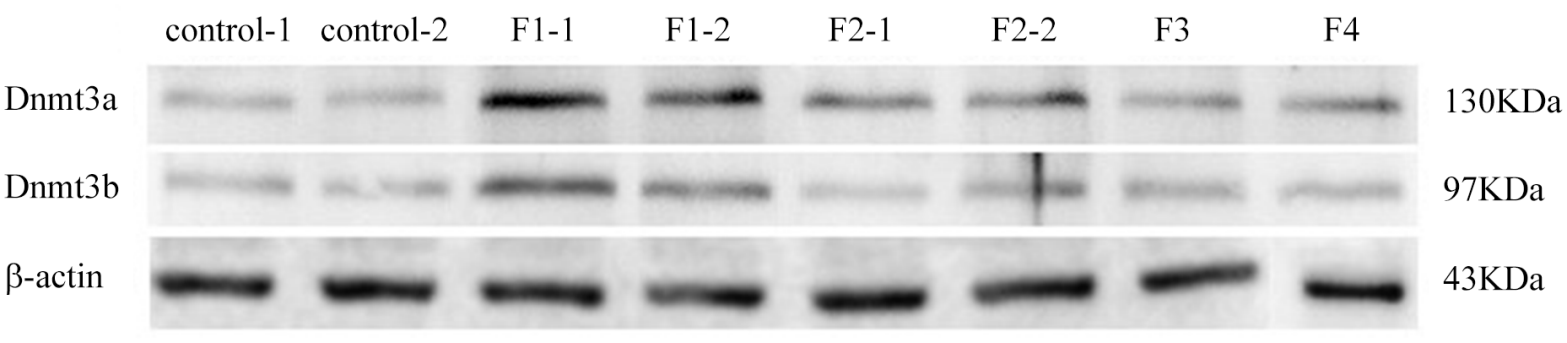
Control group and DEHP experimental group offspring (F1-F4) male rat’s DNMT3a expression. Result shows that with the increase of genetic algebra, the expression level of DNA methyltransferase gradually lowered, the testicular damage degree is reduced.

Western Blot showed similar to immunohistochemical results, F1 and F2 generations of male rats’ testis Dnmt3a expression levels were significantly higher than those of normal group;And Dnmt3b expression levels rise only in the F1, F2 and F3 generations and there is no obvious difference (Fig 7).

The result illustrates the DEHP induce offspring appeared "acquired" cryptorchidism across generations genetic is due to the change of DNA methylation modification.

**Fig 8:**
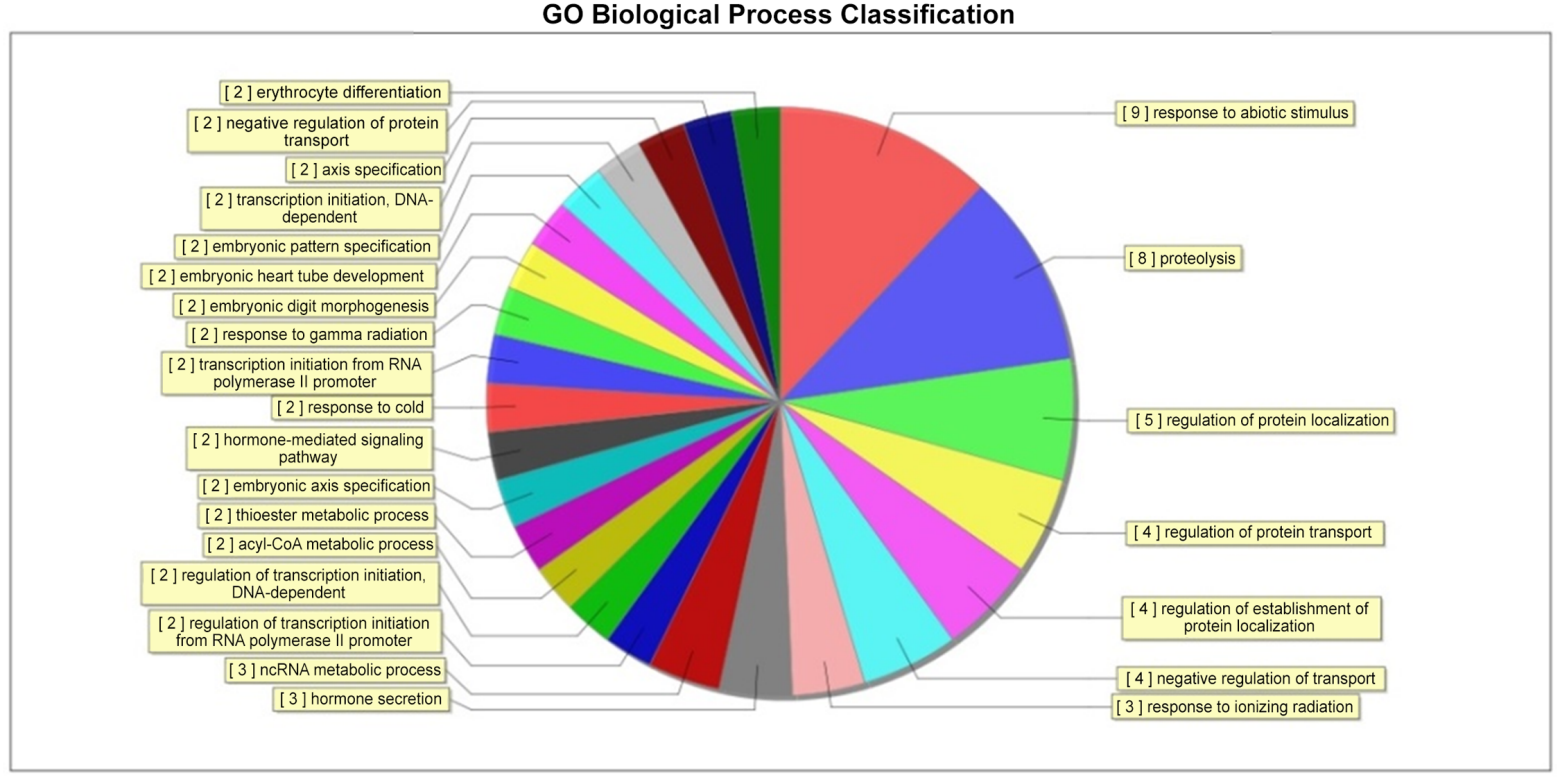
Control group and DEHP experimental group cryptochidism group compared to control group of biological process up regulation of GO Biological Process Classification.

**Fig 9:**
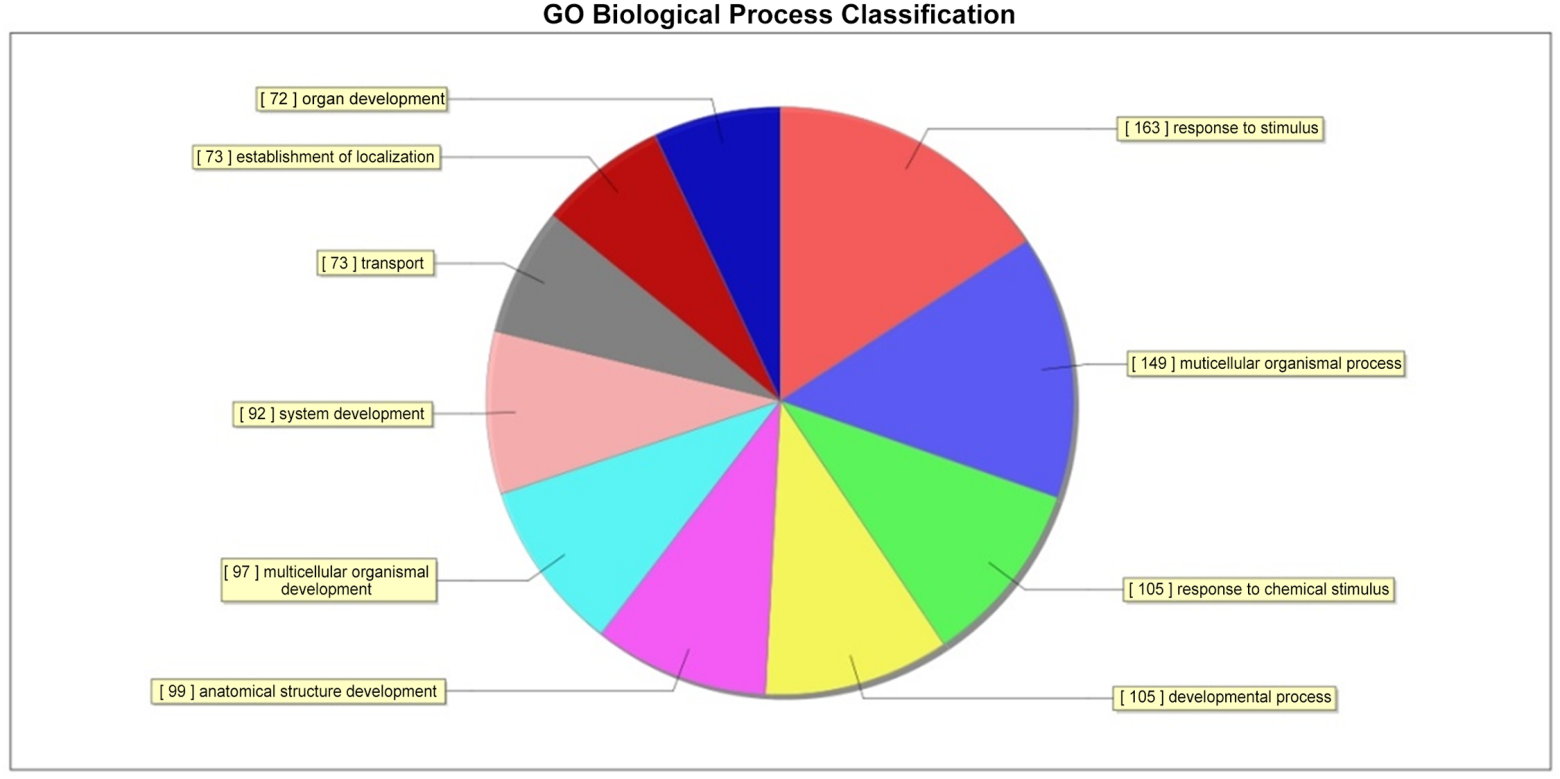
Control group and DEHP experimental group cryptochidism group compared to control group of biological process down regulation of GO Biological Process Classification.

**Fig 10:**
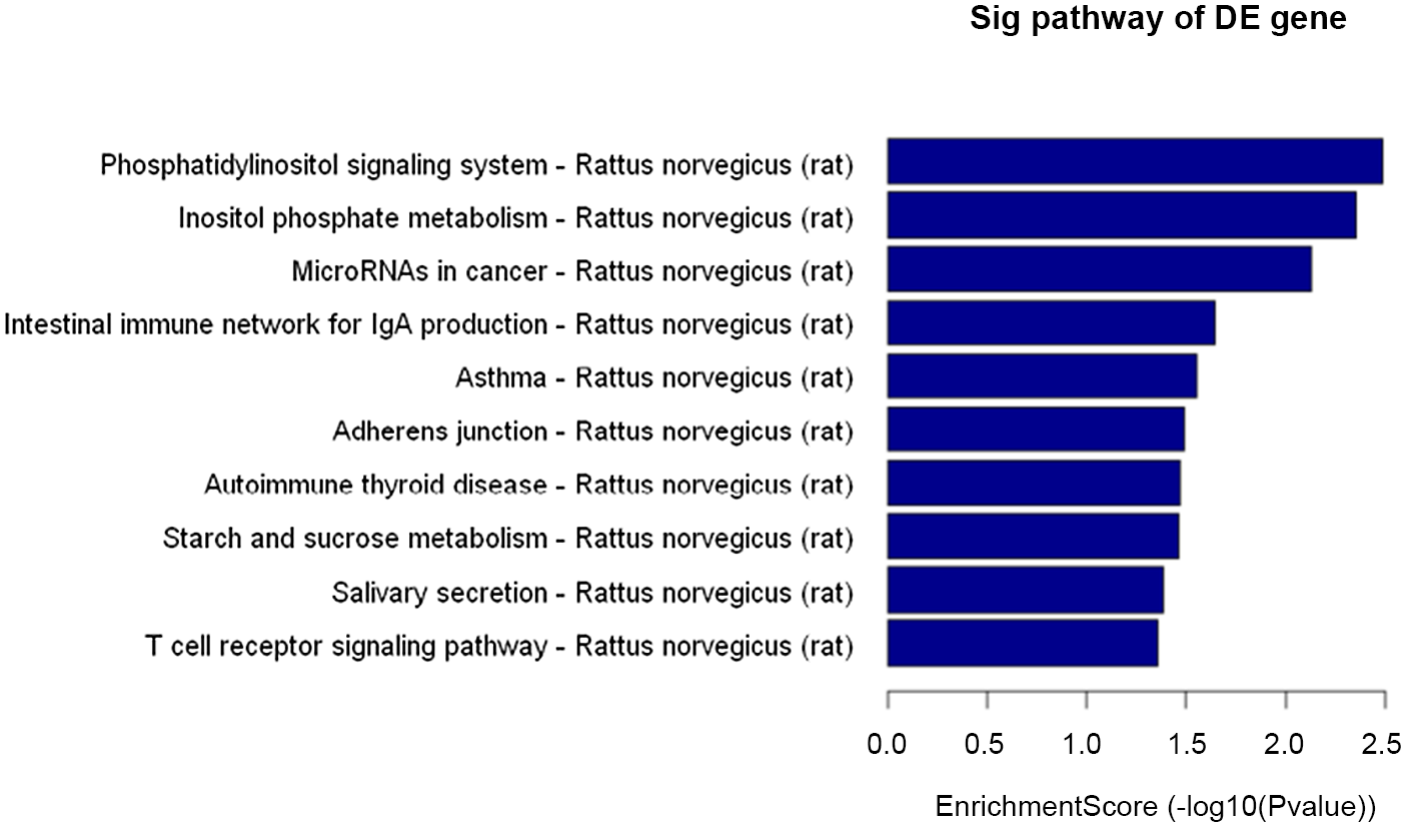
Control group and DEHP experimental group cryptochidism group compared to control group of biological process down regulation of pathway analysis.

6. RNA and DNA were isolated from the freshly rats’ testis to examine gene expression by microarray analysis and DNA methylation by methylated DNA immunoprecipitation (MeDIP) followed by analysis on a genome-wide promoter tiling array (Chip) using a comparative hybridization MeDIP-Chip analysis between control and cryptochidism samples as described in Methods. This allowed a comparison of the transcriptome or epigenome alterations in DEHP group and cotrol group. The testis DNA transcriptome analyses demonstrated that all arrays were of good quality with no abnormal hybridization detected. Differential gene expression between control and cryptochidism testes was determined as previously described. There were 103 differentially expressed genes in cryptochidism group compared to control group of biological process up regulation (Fig 8). There were 3531 differentially expressed genes of biological process down regulation(Figs 9 and 10). To calculate differentially methylated regions, we performed a Fold Change filtering between one group and one sample with MeDIP-score. The threshold is fold change > = 2.0. To calculate differentially methylated regions, we performed a t-test filtering between two groups with MeDIP-score. The threshold is fold change > = 1.5, P-value < = 0.05

In the up regulation of GO biological process, we can see differences genes have focused on response to abiotic stimulus and regulation of transcription initiation from RNA polymerase II promoter. In the down regulation of GO biological process, we can see differences genes have focused on paraxial mesoderm development and negative regulation of MAPK cascade. By pathway analysis of down regulation, the most enrichment pathway is phosphatidylinositol signaling system, there was no fingding in up regulation function.

## Discussion

Environmental factors although dosn’t cause mutations in genes or DNA sequence change,but caused by the patterns of modification of DNA methylation and other epigenetic changecould prompt response to the occurrence of diseases, more important is the "acquired" theoretically epigenetic modifications can change through the reproductive cells (gene imprinting) passed to their children, cause children's disease[13]. Epigenetics is DNA sequenceunchanged but phenotypic heritable change a discipline, its research content including DNA methylation, histone modification, non-coding RNA regulation[14], several aspects, such as any abnormal on the one hand may affect chromatin structure and gene expression of epigenetic information in the process of spermatogenesis and fertilization great changes[15]. DNA methylation modification depends on the DNA methyltransferase (DNMT DNA methyltransferase), the main members of the family of mammalian DNMT are: Dnmt1, Dnmt3a and Dnmt3b and Dnmt3L, etc. Dnmt1, Dnmt3a and Dnmt3b could catalyze methyl to the CpG dinucleotide cytosine five;Dnmt3L has no catalytic activity, but can be coordinated Dnmt3a and Dnmt3b function, is an essential factor[16]. Each member of the DNMT family in different stages have different expression patterns, respectively in methylation play an important role in the process of building and maintaining of: (1) Dnmt1 is one of the main mammals methyltransferase, expressed in the replicated state, the proliferation of cells can priority catalytic replication after half a double-stranded DNA methylation, make the lowmethylation substring fully methylation, plays an important role in maintaining;(2) Dnmt3a and Dnmt3b act in double-stranded DNA methylation, Dnmt3a priority make outside of nucleosome naked part of DNA methylation, Dnmt3b is positioned on core;(3) Dnmt3L (DNA Methyltransferase3-like Protein) in the area of PHD zinc finger with Dnmt3a and Dnmt3b homology, but the lack of highly conservative transferase motif, so there is no catalytic activity, but Dnmt3L can direct stimulation of Dnmt3a and Dnmt3b DNA methylation activity and function[2, 17].

Some kinds of synthetic chemical pollutants exist in the environment, additives by interfering with biological endocrine system affect the development and function of the urogenital system[18]. DEHP is by far our domestic consumption of the largest and the most widely used general-purpose plasticizer, a large number of used in all kinds of plastic products, such as food packaging materials, production and processing of medicinal materials, it is also the important composition of other chemical industry, through various channels to the environment caused serious "white pollution"[19], harmful to human health. Notable is DEHP toxic effects of reproductive system, not only can cause deformity, such as cryptorchidism, hypospadias, can also cause the number of sperm quality and cause infertility, is one of the most serious induced testicular cancer[7, 20, 21].

This research view point of epigenetics induced by DEHP toxicological effect, select the DEHP toxic after pregnant rats (F0), observe the F1-F4 generation of cryptorchidism, detection of DNMT expression level difference between generations. Our preliminary research results show that the changes in the level of DNA methylation may affect the growth and function of mesenchymal cells, leading to testosterone and insulin-like factor 3 (insulin-like factor3, INSL3) inadequate secretion[22], affecting testicular descent, induce the occurrence of cryptorchidism[23, 24]. Infected pregnant rat F0 give birth to F1 generation, random selected male young rats (PND80) mate with normal female to produce F2 generation, significantly increased the incidence of cryptorchidism compared with normal control group, the incidence of cryptorchidism F2 generation about 19%[4]. With the increase of generations and no more pollution, maybe the DNA repair themselves, may have happened demethylation process, make originally silence gene expression and correction[25]. Epigenetic reprogramming modifier take place in a very short window period, during the primitive germ cells in the process of migrating to genital ridge experienced completely methylation, followed by at the critical moment of sexual differentiation of genome-wide demethylation[26]. This study selected the time window is reproductive cells to methylation and methylation of critical period again. The results showed that parental (F0) DEHP exposure during pregnancy, although not in the F1 generation of mutations in genes or DNA sequence change, but can lead to the F1 DNA methylation modify state changes, three kinds of F1 generation of genome DNA methylation transferase (Dnmt1, Dnmt3a and Dnmt3b) express all have varying degrees of increase. Findings suggest epigenetics research category of DNA methylation modification model change is DEHP may have been one of the mechanisms of the F1 generation of cryptorchidism.

The latest research reports that epigenetic changes under specific environmental conditions may be multicellular organisms can be the sole basis for genetic phenotypic change[9, 27]. Genomic imprinting also known as genetic imprinting or parental imprinting, refers to control a phenotypic differences between a pair of alleles due to close to source different expression, the body only expression from one of parental alleles[2]. Genomic imprinting is a special kind of Mendelian inheritance phenomenon, namely the difference expression of alleles in the offspring from parents. Genomic imprinting is "acquired genetic" provides an interpretation: environmental change can cause the change of genomic imprinting modified model, and the imprinting modification can be passed on to the next generation through the reproductive cells[28]. DNA methylation in genome of epigenetic modifications, is an important means of regulating gene function. It changes will affect gene expression and function, resulting in abnormal gene expression and gene function abnormalities, is usually accompanied by a high methylation and gene silencing. Abnormal DNA methylation prompted C-T mutation, studies have shown that DNA methylation translate cytosine into 5-MC, and 5-MC mutation rate than other bases (including no cytosine methylation) mutation rate is high. DNMT at the same time play an important role in catalytic mutation, participation in C-U-T transformation, and so on, including the p53 tumor suppressor gene of 5-MC are hot spots of mutation in malignant tumors, abnormal DNA methylation in these mutations play a important role[29]. When the DEHP influensce organism genome DNA methylation levels, affecting the cancer gene, the change of the epigenetic modifications can lead to the inactivation of tumor suppressor genes and proto-oncogenes activation, thus inducing tumor. This experiment DEHP cause rat "acquired" cryptorchidism inter-generational genetic phenomenon, with the increase of the generations, offspring of male rat gradually reduce the incidence of cryptorchidism, male rat reproductive function damage degree to reduce gradually, increase the number of sperm, deformity rate is reduced, combined with our previous experimental results, we speculate that may be associated with genomic imprinting modification. DEHP affect genomic imprinting through DNA methylation transferase methylation modification model changes, thus inhibiting the expression of key genes, the male reproductive system development induced cryptorchidism occurred across generations.

## Conclusions

DEHP-induced changes in DNA methylation, especially within CpG islands and promoter regions, can make some key gene silence, may influence the development of male reproductive system. This changes may be passed on to the next generation, so even if the future generation is not contaminated, the same disease will happen. Epigenetic change may be one possible mechanism of DEHP-mediated testicular toxicity. Next, the role of epigenetic effects in DEHP such as cryptorchidism, requires identification of tissue-specific genes with changes in DNA methylation. The MeDIP-Chip analysis suggests an phosphorylation and negative regulation of MAPK cascade play a role in epigenetic inheritance of male reproductive. As to in which some genes play an important role, more research is needed.
