# Correction: The Mechanism of Environmental Endocrine Disruptors (DEHP) Induces Epigenetic Transgenerational Inheritance of Cryptorchidism

**DOI:** 10.1371/journal.pone.0132749

**Published:** 2015-07-06

**Authors:** Jinjun Chen, Shengde Wu, Sheng Wen, Lianju Shen, Jinpu Peng, Chao Yan, Xining Cao, Yue Zhou, Chunlan Long, Tao Lin, Dawei He, Yi Hua, Guanghui Wei

There are errors in the Funding section. The correct funding information is as follows: The project was supported by the National Natural Science Foundation of China (grant numbers: 81370701), the National Clinical Key Subject Construction Project (No.: AXA medical office letter [2013]544), the 2013 Program for Innovation Team Building at Higher Education Institutions in Chongqing, China and the special fund of Chongqing key laboratory (CSTC).
